# Health and Nutrition Promotion Programs in Papua New Guinea: A Scoping Review

**DOI:** 10.3390/nu16131999

**Published:** 2024-06-24

**Authors:** Juliana Chen, Alyse Davies, Phyllis Tran, Ryley Gronau, Anna Rangan, Margaret Allman-Farinelli, Shelina Porykali, Robin Oge, Bobby Porykali

**Affiliations:** 1 Discipline of Nutrition and Dietetics, Susan Wakil School of Nursing and Midwifery, Faculty of Medicine and Health, The University of Sydney, Sydney, NSW 2006, Australia; alyse.davies@sydney.edu.au (A.D.);; 2 Charles Perkins Centre, The University of Sydney, Sydney, NSW 2006, Australia; 3 School of Health Sciences and Social Work, Griffith University, Gold Coast, QLD 4222, Australia; 4 National Capital District Provincial Health Authority, Port Moresby 121, Papua New Guinea; 5 Aboriginal and Torres Strait Islander Health Program, George Institute for Global Health, Sydney, NSW 2000, Australia; bporykali@georgeinstitute.org.au; 6 Faculty of Medicine, School of Population Health, University of New South Wales, Sydney, NSW 2052, Australia

**Keywords:** diet, nutrition, non-communicable disease, communicable disease, health promotion programs, mHealth, Papua New Guinea, Pacific Islander, Healthy Islands

## Abstract

There is a rising prevalence of non-communicable diseases (NCDs) in Papua New Guinea (PNG), adding to the disease burden from communicable infectious diseases and thus increasing the burden on the healthcare system in a low-resource setting. The aim of this review was to identify health and nutrition promotion programs conducted in PNG and the enablers and barriers to these programs. Four electronic databases and grey literature were searched. Two reviewers completed screening and data extraction. This review included 23 papers evaluating 22 health and nutrition promotion programs, which focused on the Ottawa Charter action areas of developing personal skills (12 programs), reorienting health services (12 programs) and strengthening community action (6 programs). Nineteen programs targeted communicable diseases; two addressed NCDs, and one addressed health services. Enablers of health promotion programs in PNG included community involvement, cultural appropriateness, strong leadership, and the use of mobile health technologies for the decentralisation of health services. Barriers included limited resources and funding and a lack of central leadership to drive ongoing implementation. There is an urgent need for health and nutrition promotion programs targeting NCDs and their modifiable risk factors, as well as longitudinal study designs for the evaluation of long-term impact and program sustainability.

## 1. Introduction

Papua New Guinea (PNG) is a linguistically, ethnically, culturally and geographically diverse Pacific Island Nation, having over 800 living languages, including three national spoken languages of English, Tok Pisin and Hiri Motu, and consisting of four distinct geographical regions, namely the Highland region, Southern region (including Port Moresby, the Capital of PNG), Momase region, and New Guinea Islands region. The diversity and isolation of geographic regions and a large number of cultural groups historically have made it hard to govern since its independence from Australia in 1975 [1], but also made it challenging to develop health promotion initiatives, communication and policies for health for the whole country [1].

The population of PNG face the dual burden of communicable and non-communicable diseases (NCDs), with the country undergoing an epidemiological shift from infectious diseases to NCDs [2]. While mortality from infectious diseases such as tuberculosis (TB), malaria, and human immunodeficiency virus (HIV) still make up 35% of total deaths, at the same time, mortality from NCDs, such as cardiovascular diseases (CVD) and type 2 diabetes mellitus (T2DM) has increased dramatically [2]. In 2018–20, NCD-related deaths in PNG accounted for 47% of total deaths, showing a 10% increase over the past 50 years [2]. This places significant pressure on the already compromised healthcare system with added socioeconomic burden and negative effects on individual and community health [3].

As with other Low to Middle-Income Countries (LMIC) across the Pacific, PNG is undergoing rapid economic growth, social transition and urbanisation due to increasing resource development [4]. This has coincided with the nutrition transition phenomenon whereby the population’s traditional diet and physical activity behaviours have shifted to more processed, high-energy dense foods, increased dependence on global food supply and sedentary activity [4]. Similar to other Pacific LMICs, where the economic and nutrition transition have been associated with increased NCD prevalence [5,6], in PNG, the nutrition transition is more apparent in regions with higher exposure to urbanisation and modernisation and therefore, the peri-urban and urban populations are at higher risk of NCDs such as CVD and T2DM compared to their rural counterparts [7,8]. 

Communicable diseases have been the focus of many health promotion programs in PNG, aimed at delivering education to increase awareness of infectious diseases and reduce the burden of disease [9,10,11,12]. This included initiatives such as the Women and Children’s Health Project carried out in PNG between 1998 and 2004 [13], as well as other local health campaigns focusing on TB and malaria [9,10]. The National Policy on Health Promotion for PNG aims “to empower individuals and communities thereby enabling them to control the status of their own health”; however, the policy recognised a gap in health promotion activities specifically for ‘Healthy Islands’—a vision encouraging preventive measures for health promotion and health protection [14].

There are renewed efforts from the PNG government as part of the 2021–2030 National Health Plan and ‘Healthy Islands’ concept to focus on health promotion, including nutrition promotion [15]. The plan prioritises, as part of its strategies for preventing and reducing the morbidity and mortality of NCDs, increasing population awareness so that individuals can make more informed decisions about their health and engaging in collaborations with stakeholders to implement nutrition programs [15]. Furthermore, with survey findings revealing smoking, being overweight or obese, and impaired fasting glycemia to be among the common NCD risk factors in PNG for those aged between 15 and 64 years [16], health promotion interventions targeted at lifestyle modification, including diet and exercise could support the management and reduction of NCD-risk.

Therefore, this scoping review aimed to scope the body of literature for health and nutrition promotion programs that have been implemented in PNG, including both communicable diseases and NCDs, and determine the enablers and barriers to these programs [17]. These findings could then be used to inform future health promotion programs, particularly for preventing and managing NCDs in PNG. 

## 2. Materials and Methods

### 2.1. Protocol and Registration

The protocol for this scoping review was registered on https://osf.io/pvkg8/ (accessed on 18 June 2024). The findings are reported in accordance with the PRISMA extension for scoping reviews, and Joanna Briggs Institute updated methodological guidance for scoping reviews [18,19].

### 2.2. Eligibility Criteria 

The inclusion and exclusion criteria have been summarised in Table 1.

#### 2.2.1. Type of Evidence Sources 

All primary study designs, government reports, and websites were included. Conference abstracts, theses, systematic reviews, meta-analyses, policy papers, expert opinions and non-peer-reviewed articles were excluded. Studies were limited to human studies, and language was restricted to English. There was no date of publication limit applied to capture all health promotion programs that had been conducted in PNG over time.

#### 2.2.2. Participants and Context

All ages, regions, and settings of PNG (primary health care, schools, workplaces, churches or community centres) were included. Studies based on alcohol or drug rehabilitation, live-in facilities, and facilities for diagnosed mental illness were excluded.

#### 2.2.3. Concept

Primary health promotion interventions that focused on the prevention of NCDs, including over and undernutrition, infectious diseases including malaria, TB and sexually transmitted diseases including HIV and the Human papillomavirus (HPV), as well as sexual health were included. While it is recognised that health promotion policies such as healthy catering policies, no smoking policies, environmental policies, or urban design modification will often support health promotion interventions, these were excluded from this review, given the focus placed on interventions. Papers solely reporting on recommendations for future health programs were excluded. 

### 2.3. Information Sources

Relevant studies were identified through a comprehensive literature search using the following electronic databases: Medline, Embase, Global Health, and Scopus. Grey literature searches were conducted by using the advanced search function on Google Scholar (limited to the first 200 records). A citation search of articles included for full-text screening was performed to scan for additional documents. 

### 2.4. Search Strategy and Terms

The review team drafted the search strategy in consultation with an experienced university librarian (M.C.). See Supplementary Table S1 for the final MEDLINE search strategy. The final search for all databases was conducted in March 2023.

### 2.5. Study Selection Process

All documents retrieved from the search were exported to EndNote20 citation management software to remove duplicates. The citations were imported into Covidence (Veritas Health Innovation, Melbourne, Australia) to be independently screened by two reviewers (P.T. & R.G.) in accordance with the eligibility criteria. The study selection process was conducted in two stages. The first stage involved independent screening of titles and abstracts by two reviewers against the inclusion criteria. The second stage involved independent screening of full texts by two reviewers against the inclusion and exclusion criteria. For both stages, any discrepancies between the two reviewers were resolved by a third reviewer (J.C., A.D. or B.P.).

### 2.6. Data Extraction and Charting Process

Data extraction was conducted independently by two reviewers (P.T. & R.G.) using a standardised data charting form for scoping reviews [18]. The following data were extracted: first author and year, program/intervention name, aim, targeted communicable disease or NCD, setting, participants, study design, a brief description about the program, the Ottawa Charter key action areas for Health Promotion (i.e., create supportive environments for health, strengthen community action for health, develop personal skills, and reorient health services) [14,20] addressed by the program, enablers and barriers to the programs and the main findings. 

### 2.7. Synthesis of Results

The results were presented in tabular form, with an accompanying narrative summary to describe the results in relation to the objectives of the scoping review.

## 3. Results

**Figure 1:**
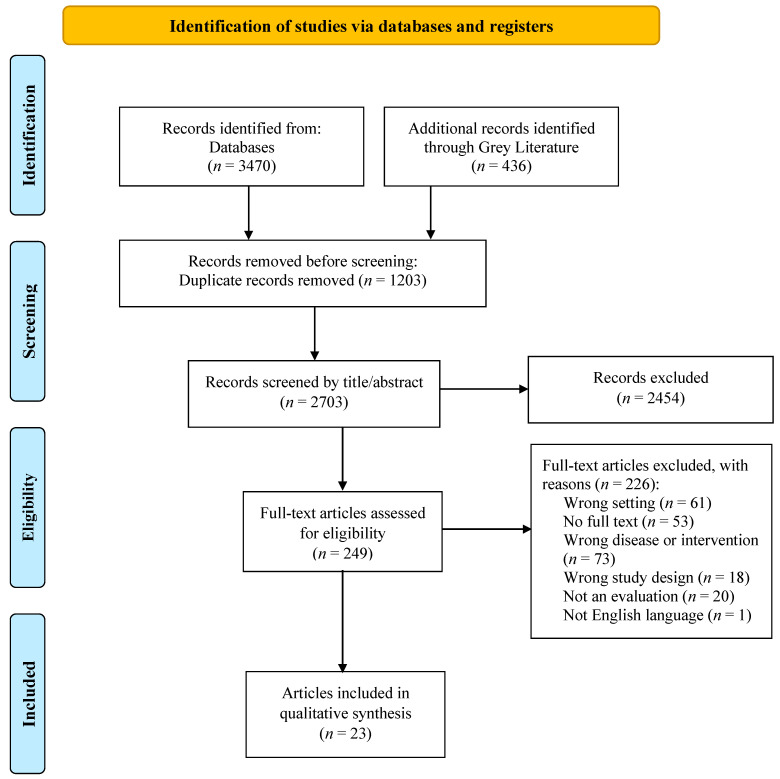
PRISMA-ScR flow diagram of record identification and study selection for a scoping review on health and nutrition promotion programs conducted in PNG [21].

This scoping review yielded a total of 3906 records from databases, grey literature and citation searching. Following the removal of 1203 duplicates, 2703 records were screened by title and abstract. A total of 249 records underwent full-text screening, and 226 records were excluded with reason, resulting in a total of 23 articles meeting the inclusion criteria and included in this review. The PRISMA-ScR flow diagram illustrates the selection process (see Figure 1). 

### 3.1. Program Selection and Characteristics 

Study and program characteristics are described in Table 2. Of the total 23 articles included, there were 17 peer-reviewed articles [22,23,24,25,26,27,28,29,30,31,32,33,34,35,36,37,38], five grey literature reports [39,40,41,42,43], and one book chapter [44]. Five were quantitative studies [23,29,30,39,40], 13 were qualitative [22,24,25,28,31,32,33,34,35,36,42,43,44], and five were mixed-methods [26,27,37,38,41]. The included publications (1996 to 2022) spanned over a period of 26 years.

**Figure 2:**
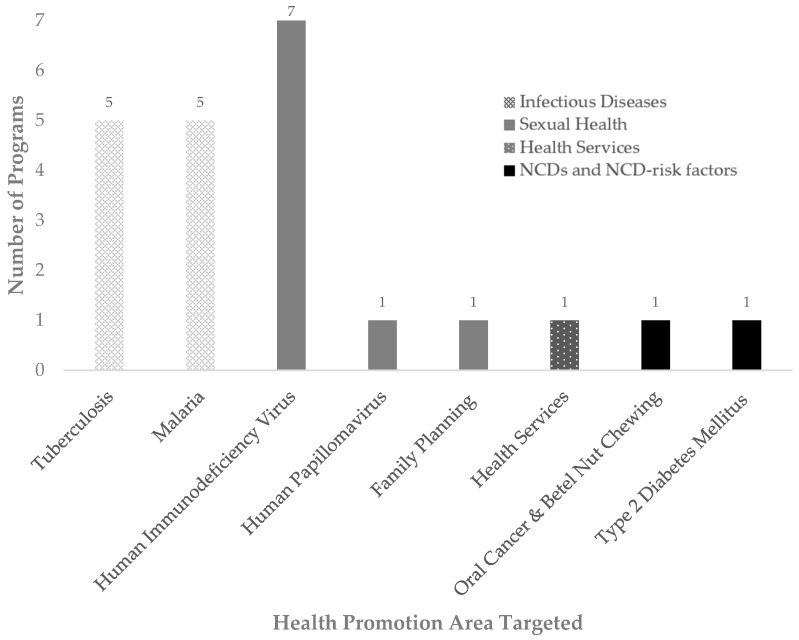
The different health promotion areas targeted by the programs.

A total of 22 programs focusing on health promotion interventions in PNG were identified from 23 articles. Of these 22 programs, 19 programs targeted communicable diseases (see Figure 2). Ten programs targeted infectious diseases, TB (*n* = 5) [22,23,39,40,41] and malaria (*n* = 5) [24,25,26,27,28]. Nine programs targeted sexually transmitted diseases and sexual health, HIV (*n* = 7) [29,30,31,32,33,42,43,44], with three specifically targeting parent-to-child HIV transmission [29,30,31], HPV (*n* = 1) [34], and one on family planning (*n* = 1) [35]. One health promotion program addressed health services [36]. Two programs targeted NCD-risk factors and NCDs, namely betel nut chewing [37] and a nutrition promotion program on T2DM [38].

Four programs took place at a national level across PNG [22,23,26,35], and nine programs were delivered at a community level across PNG [32] and in different regions of PNG, including the Highlands [24,25,33,39,43,44], Highlands, Momase and Southern PNG [42], and New Guinea Islands [38]. Eight programs were in the health setting, targeting health workers or people attending hospitals and/or health service clinics or facilities across PNG [28,36] as well as specifically in New Guinea Islands [40], Southern [41], Highlands [27,29], Southern and Highlands [30,31], and Momase and Highlands [34]. One program was in the school setting (Momase [37]). 

### 3.2. Program Aims

**Figure 3:**
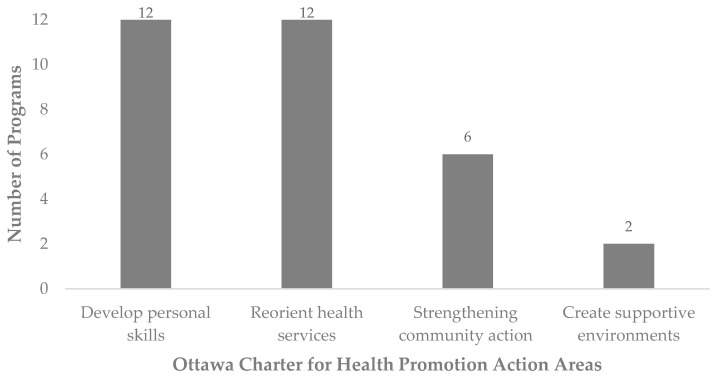
The Ottawa Charter for Health Promotion action areas targeted by the programs.

The programs aimed to either control, prevent or reduce the disease burden of TB, malaria, HIV, HPV, betel nut chewing and T2DM through different health-promoting strategies, which could be categorised by the following key action areas of the Ottawa Charter for Health Promotion (Figure 3):

#### 3.2.1. Develop Personal Skills 

Twelve programs focused on developing personal skills. Ten programs incorporated a component of training, education or awareness raising as a strategy to spread knowledge about the disease for increased prevention, screening or treatment [24,25,33,34,37,38,39,40,42,43,44], and three programs focused on the provision of counselling and care [29,30,39].

#### 3.2.2. Reorient Health Services

Twelve programs addressed the health promotion action area of reorienting health services. Two programs included training health workers to provide better care or education to the public [34,40]. There was one training program for health workers to become field epidemiologists to strengthen the public health workforce and health systems [36]. Seven programs involved screening, treatment or diagnosis, including surgical or medication programs [22,23,29,30,31,35,41]. There were two programs that used technology for malaria control, such as m-health for health data surveillance or text messaging reminders to healthcare workers [27,28].

#### 3.2.3. Strengthening Community Action

Seven articles reporting on six programs were targeted at strengthening community action. This was observed in the mobilisation of community members to take action, either through working together to create health-promoting messages, training for diagnosis and treatment, or peer counselling for other community members [22,24,25,32,39,43,44].

#### 3.2.4. Create Supportive Environments

Two malaria programs sought to ‘create supportive environments’ by providing bed nets for malaria prevention [25,26].

### 3.3. Enablers to Health and Nutrition Promotion Program

Factors that enabled the program were cultural appropriateness, community involvement, educators, resources, staff and funding, and health service delivery, described below and in Table 3.

#### 3.3.1. Cultural Appropriateness and Adaptation

A common characteristic influencing program effectiveness was understanding cultural context to ensure cultural appropriateness [24,25,29,32,33,34,35,38,44]. In the Batri Village Bed Net Initiative for the provision of nets to a rural community, the villagers felt respected as the program was inclusive and authentic, which could be attributed to the incorporation of language-translation tools and prior communication with village elders [25]. In an HIV prevention program, local communities were engaged in a bottom-up approach to build capacity and support local knowledge, storytelling, interests, customs, and languages in order to achieve changes in attitudes, behaviours, and social norms [32]. In another education program for HIV, village leaders were involved in decisions about the education session to determine the appropriateness of the content for community cooperation and enthusiasm [33].

Improved knowledge of T2DM was seen after carrying out a nutrition promotion program that was culturally sensitive and interactive and utilised a storytelling model tailored to Indigenous communities in the New Guinea Islands [38]. An interpreted supported translation into a local dialect. The program involved a ‘Sugarman’ presentation designed to raise awareness about choosing local non-processed foods commonly consumed by the Bougainvillean population over high-caloric Western foods and their impact on health, particularly in relation to T2DM. The program emphasised T2DM prevention and management through healthy lifestyle choices such as exercise, a healthy diet, and not smoking.

#### 3.3.2. Community Involvement

Another factor in programs with positive impact or community acceptability was involving the community in the programs in different ways. Community involvement in helping to counsel and educate other members of the community contributed to reduced loss to follow-up for TB care [39]. Training women for medicine storekeeping and quality assurance of diagnosis in a community with high rates of malaria helped to reduce the number of cases [24]. Community participation in the innovative creation of health messages and interventions through different media, including visual and digital media, helped to create localised meaning by tailoring the content to their own culture, language and literacy levels [32,43,44]. This allowed the communities to be more readily accepting of the messages. 

#### 3.3.3. Educators

In an education program, it was important to train educators to be flexible in different teaching methods, responsive to community concerns, and able to foster a comfortable environment when talking about potentially embarrassing topics, as this was effective in ensuring participant understanding and dialogue [33]. Healthcare workers who were knowledgeable and understanding when providing education created a trusting patient-provider relationship, which was a major factor in the effectiveness of the HPV program [34]. 

#### 3.3.4. Resources, Staff and Funding

Mobilisation of funding and resources, including technology and medicines, contributed to the effectiveness of TB and HIV treatment programs [23,30,31,41].

#### 3.3.5. Leadership and Partnership Collaboration

Strong government commitment or leadership in conjunction with effective collaborations between technical partners, NGOs, community and government contributed to increased treatment rates, training and research programs, particularly with regards to increased sustainability of the program [23,35,36,41].

#### 3.3.6. Health Service Delivery

Health workers reported that the incorporation of mobile health (mHealth) technologies, like mobile phone text message reminders and mHealth apps linked to health professional platforms via geographical coding, were helpful in reinforcing health service delivery messages and monitoring outbreaks [27,28]. mHealth reminders were also useful for reminding patients of their appointments to improve patient compliance with attending TB treatment [40]. Decentralisation of care for TB health services to the district level increased screening and reduced unfavourable treatment outcomes [40].

### 3.4. Barriers to Health and Nutrition Promotion Program

Lack of cultural appropriateness; resources, staff and funding; leadership and health service delivery; and lack of behaviour change campaigns were several common characteristics that emerged as barriers to the programs (see also Table 3):

#### 3.4.1. Lack of Cultural Appropriateness and Adaptation

Some programs did not consider language and cultural context, which caused problems in a low literacy setting. For example, print media for HIV awareness were in English, yet there was an overwhelming preference for Tok Pisin to be used in most covered regions [42]. In Milne Bay Province, there was a preference for one or two of the four dominant tok plus languages (local language) [42]. Knowledge outcomes of a betel nut chewing education program were not significantly improved for primary school students, likely due to English being a second language for most [37]. 

#### 3.4.2. Resources, Staff and Funding

A common dissatisfaction with treatment programs was a lack of sufficient supply of medications in health services [22,29]. Additionally, lack of treatment supervision due to a lack of staff or service scope led to unfavourable treatment outcomes or increased loss to follow-up rates [22,31]. Lack of resource supplies such as bed nets led to unfavourable outcomes for malaria [26].

#### 3.4.3. Leadership and Health Service Delivery

The lack of central leadership and integration of health services led to poor delivery of health service-based programs [31,35]. 

#### 3.4.4. Cost

The cost of attending health services, including transport and treatment costs, contributed to a loss in follow-up rates, particularly for those living in rural areas [31]. 

#### 3.4.5. Lack of Behaviour Change Campaigns

A resource provision program for mosquito bed nets did not see favourable outcomes as it was not accompanied by behaviour change or awareness campaigns to encourage increased use of the bed nets [26]. 

### 3.5. Program Outcomes

#### 3.5.1. Tuberculosis

Programs that incorporated training, education, counselling, and awareness were generally able to increase screening and retention in care. However, this did not necessarily lead to significant improvement in health outcomes [39,40]. The national TB treatment programs had mixed findings across PNG [22,23,41].

#### 3.5.2. Malaria

Training community members to diagnose and treat malaria for others in the same community, as well as community-level net provision programs that were integrated with education sessions, helped to lower malaria mortality and morbidity [24,25]. The nationwide net provision program also increased ownership and usage of nets to prevent malaria [26].

#### 3.5.3. Sexually Transmitted Diseases

For parent-to-child HIV programs, there was improved HIV-free survival with time as programs incorporated more resources and a more family-centred approach; however, loss to follow-up rates remained unacceptable, and efforts towards increasing health service accessibility are necessary [29,30,31]. A community-level program involving social mobilisation for HIV demonstrated promising signs of changing community attitudes and behaviours [32]. HIV education and awareness programs through visual media or education sessions were positively received, with most participants understanding the importance of screening, changing their attitudes towards people living with HIV and identifying individual preventative behaviours [33,42,43,44]. Health outcomes for HIV programs were not reported [29,30,31,32,33,42,43,44].

The national no-scalpel vasectomy program for family planning had mixed outcomes throughout the country [35].

The pilot HPV screening and treatment program showed a high level of acceptability for PNG women, which could potentially increase future screening uptake [34].

#### 3.5.4. Non-Communicable Diseases

The education program for school children about NCD risk factors (betel nut chewing) showed an improved understanding of the topics for most participants; however, there was no significant change in knowledge of betel nut chewing for primary school children [37]. The Sugarman presentation improved understanding of the causes, symptoms, and prevention of T2DM among communities, with the greatest improvement being in communities where there was initially poor knowledge about T2DM [38]. However, health outcomes, such as anthropometry or biochemistry, were not measured or reported for T2DM or betel nut chewing [37,38].

## 4. Discussion

This scoping review is the first to synthesise evidence from 22 health and nutrition promotion programs (23 articles) in PNG and to identify factors that were enablers and barriers to these initiatives. Most health promotion programs (*n* = 20) in this review targeted communicable diseases, particularly infectious diseases. There was only one health promotion program targeting NCD risk via betel nut chewing and oral cancer [37] and one nutrition promotion program for T2DM [38]. Both programs demonstrated improvements in participant awareness of strategies for preventing these NCDs, though the impact on health was not measured. Enablers of health promotion initiatives included cultural appropriateness, mobilising community action and using mHealth technologies to decentralise health services. On the other hand, many programs were unsustainable or resulted in unfavourable outcomes due to lack of resources, funding and ineffective leadership for sustained implementation. The paucity of health and nutrition promotion programs targeting diet and modifiable lifestyle risk factors highlights the urgent need to prioritise the delivery and evaluation of NCD-related health promotion.

Directly addressing the NCD burden and targeting health priorities has been emphasised in the PNG National Health Plan 2021–2030 and policy directions [15]. The priority of revitalising the Healthy Islands approach for health promotion and prevention efforts through healthy lifestyle behavioural modifications to support better health [15] is also timely. While in our review, there were few health and nutrition promotion programs in PNG targeting NCDs, enablers to health promotion programs targeting communicable diseases could be adopted into future health and nutrition promotion programs for NCDs. Foundational to Healthy Islands is an underpinning of community participation and empowerment activities [14]. For community-level programs, understanding contextual elements is essential to the development of effective behaviour change interventions [45]. Health programs in PNG that incorporate active community involvement to ensure cultural appropriateness, better understanding of the cultural and social milieu as well as cooperation and collaboration between participants and educators or researchers, demonstrated positive outcomes [25,32,33,38,43,44]. By providing information in local languages or the common language, Tok Pisin, health promotion programs can overcome language and literacy barriers and ensure that messages are culturally appropriate and relevant. Particularly, the involvement of village leaders or influential community figures in the decision-making process may help to build trust, and therefore, communities may be more readily accepting of the health messages [33]. This is especially important in settings such as PNG, where there can be a distrust of ‘outsiders’ when conducting health programs [46]. Hence, developing interventions and health promotion programs that are embedded in co-design with communities will ensure that delivery, ownership and acceptance of these initiatives are sustainable and affordable. A holistic approach to health, addressing physical, mental, social and spiritual well-being, is essential when working with communities in PNG. Being a Christian nation, churches and religious leaders are highly respected and can, therefore, be an integral component in allowing health promotion programs to provide education, spread health information, and deliver health care services to communities [47,48]. The importance of including churches and religious leaders is also highlighted in the framework for Pacific health research and from work in Pacific communities in New Zealand [49].

Analyses have suggested that PNG has experienced slower effects of the nutrition and epidemiological transition [50,51] compared to other Pacific Island Countries and Territories (PICTs). However, poor data precludes robust estimates of NCDs in PNG and larger population numbers in PNG relative to other PICTs, which means the nutrition transition impacts a wider subset of the Pacific population. While there are few health and nutrition promotion programs for NCDs in PNG, lessons could be drawn from health promotion programs that are available and implemented in other PICTs. For example, a Fijian health promotion initiative, the Healthy Youth Healthy Communities project, targeted adolescent obesity and NCD prevention through improving nutrition and physical activity behaviours [52]. The program considered holistic elements of the Ottawa Charter’s key action areas through school-based nutrition policies, education and role play, social marketing, the creation of supportive environments through water fountain installation, community capacity building, empowerment, and collaboration. Outcomes revealed a reduction in body fat percentage but mixed behaviour change results [53]. In a systematic review by Palu et al. [54], lifestyle-based health and nutrition promotion programs among populations in PICTs (though predominantly conducted in New Zealand) were found to be efficacious for weight loss. Particularly, the study also emphasised the importance of culturally conducive approaches, community action, peer support, and behaviour change strategies as important factors in intervention design, which is consistent with the enablers from programs targeting communicable diseases identified in our review.

The PNG government have acknowledged that adopting digital health interventions is an innovative means for supporting citizens in improving their education with mobile technologies, such as smartphone applications (apps) and e-learning [15]. In Western countries, digital platforms and mHealth apps have been found to have potential as nutrition promotion tools for modifying lifestyle behaviours like physical activity and sedentary time, as well as improving nutritional knowledge and self-efficacy [55]. They have also been found to support the management of NCDs [56,57,58] and NCD-risk factors [59]. The potential of mHealth technologies in developing countries or LMIC has also been documented for improving health outcomes for infectious diseases and maternal health [60], for health promotion of communicable diseases and NCDs [55] and effective in promoting physical activity and healthy diets [61]. Evidence of the adoption of mHealth technologies into health promotion programs for communicable diseases was observed in this review. Three programs incorporated mHealth technology (text messaging and apps) to support and reorient health service delivery, which improved patient attendance at health appointments, aided health workers in education about treatment and enabled the monitoring of disease outbreaks via geographical coding [27,28,40]. This is consistent with another scoping review by Van Olmen et al. [62], which reported that across LMIC, text messaging was the main mHealth medium applied, particularly for patient-centred NCD self-management. However, notably, in Van Olmen et al.’s review, no PICTs were included as part of the LMIC [62]. When examining lifestyle intervention for weight loss and NCD-risk factors in PICTs, no studies included any mHealth technologies [54]. Therefore, there is potential for PNG health and nutrition promotion programs to adopt mHealth technologies. However, some key considerations when using mHealth technologies in PICTs and PNG are improving digital and digital health literacy, device accessibility, internet connectivity and infrastructure for mHealth use and developing strategies to maintain ongoing engagement with mHealth technology over time to facilitate behaviour change.

Partnerships with local governments, non-governmental organisations, and other partnering countries for leadership to secure funding and human resources are essential for ensuring that health promotion programs are sustainable and successful. It is important to consider that in PNG, foreign aid from other countries or non-government organisations is a major funding source for health promotion activities. Taking vaccination programs in PNG as an example, which have been successful, UNICEF has supported the PNG government by purchasing vaccines and helping to maintain the cold chain system by working with the 22 provincial health authorities to deliver standardised programs across the country [63]. Australian Aid (AusAID) likewise has supported the vaccination of close to 1 million children in PNG since 2009 [64]. World Health Organisation (WHO) also supports the PNG government by providing mostly technical assistance [65]. Together, a unified effort from various sectors, reinforced by funding and resource allocation, is therefore crucial for the sustainable delivery of health promotion programs.

The results of this review indicated that health promotion initiatives only addressed one or two of the action areas outlined in the Ottawa Charter for Health Promotion. Comprehensive approaches to health promotion are therefore required to ensure that disease prevention and improving health and quality of life are addressed at all levels, individual, community, and national, to support healthy behaviours and provide an enabling environment. Building healthy public policies that drive health and nutrition promotion programs is an area requiring further investigation, with a particular focus on the evaluation of the impact of these policies. Evaluations of PNG national policies and strategic plans have been undertaken for TB and malaria control, NCD preventative policies have been monitored, and nutrition has even been improved [66,67,68,69]. The evaluation of NCD policies in PNG found that food and healthy eating policies, in particular, were underdeveloped, while preventative policies around tobacco, alcohol and physical activity had a higher presence and strength of implementation [68]. The evaluation of PNG’s National Nutrition Policy Strategic Action Plan (2018–2022) [70] found that the main challenge of implementing the nutrition policy and action plan included the absence of political and senior management leadership, lack of funding, resources and staffing, leading to limited roll-out of activities related to this policy and plan as well as limited monitoring and evaluation [69].

The barriers to policy implementation are consistent with many of the barriers to health promotion program implementation in PNG, as seen in this review. Therefore, it is suggested that for health promotion policy and strategy implementation, there must be a high-level political commitment to raising awareness among politicians about the importance of emerging health issues [69,71]. These challenges may be a reflection of structural health system issues since the decentralisation of health service delivery to the provincial level following PNG’s independence in 1975 [47]. This is consistent with a recent case study of the PNG healthcare system, which stated ineffective governance, systemic issues with provincial financing, insufficient resources and health workers, and deteriorating infrastructure as some of the key barriers to primary health care [47]. A coordinated and collaborative approach with political leadership at the national and sub-national levels, health promotion and nutrition committees, advisory groups and stakeholders are thus required for advocacy and to support the implementation of health and nutrition promotion activities at community-based provincial and district levels and to subsequently generate evidence for the policies themselves [69].

### 4.1. Future Directions and Recommendations

Knowledge about nutrition and NCDs in the community is poor [48]. Therefore, future recommendations to combat the emerging health issues in PNG, as described in this review, include the development of health promotion programs targeting nutrition and physical activity. Programs should adopt a systematic and multilayered approach targeting individuals, communities and the wider public. This type of collective approach has been found to be effective in research on chronic diseases and behaviour change with Pacific communities [49] and emphasised by Pacific health research guidelines [72]. Health promotion programs should focus on long-term sustainability through multi-faceted strategies that include education components and the involvement of key community figures in the development process. For instance, programs aimed at increasing education and awareness, such as the T2DM program in Bougainville, should not only seek to increase the participants’ knowledge of diabetes [38] but also be inclusive of families and relatives and the wider community, such as by utilising church groups.

Community-based participatory research approaches should be used in the design of future health and nutrition promotion programs so that health messages and activities can be co-designed with communities. Drawing from successful COVID-19 vaccination, immunisation and family planning activities programs in PNG, the involvement of village or community leaders and participants in health promotion activities is fundamental to the adoption and uptake by communities. Considering the culturally diverse landscape of PNG, an emphasis should be made in all health and nutrition promotion program planning to ensure cultural appropriateness and adaption of program activities to each specific region and local community, for example, using the language for program delivery and resources, or tailoring the types of foods recommended in nutrition promotion program, given the variability in diets across the different PNG regions [73]. Furthermore, in a culture where there are strong oral traditions, greater incorporation of cultural storytelling into programs may also enhance future program outcomes in this context [74], as there was only one study in this review which utilised this approach [38].

Based on our findings, it is recommended that funding and aid bodies (e.g., AusAID, Australian Department of Foreign Affairs and Trade, UNICEF, WHO) should also ensure appropriate cultural considerations are evidenced as part of their funding criterion in aid and project work undertaken in PNG and the Pacific more broadly. Locally, governments should consider continued support through funding for the expansion or ongoing delivery and tailoring or modification of successful programs, such as education programs that improve health literacy in specific social and cultural contexts throughout PNG, particularly as the population of PNG has low literacy levels around the emerging NCDs [75].

Drawing from obesity prevention programs in similar settings, in conjunction with health promotion programs that empower the community, it is critical to also implement and monitor compliance with health and nutrition policies, such as PNG’s 2021-2030 National Health Plan. This could include more widespread dissemination of the Pacific Guidelines for Healthy Living [76,77] through channels such as schools, churches, workplaces, or health services, which may improve population awareness of methods for reducing NCD risk. The guidelines address ten areas, including a healthy diet, physical activity, betel nut chewing and other drugs, and community involvement. These resources would also need to be embedded with further behaviour change initiatives and in-person programs run in local communities to make the public more aware of the resources and how to practically implement them in everyday life. Furthermore, multi-sectorial and muti-faceted approaches that address structural factors of rapid urbanisation and marketing of unhealthy foods will also encourage preventative behaviours and potentially better health outcomes for the people of PNG [78].

With the inconsistency in measures of program outcomes reported across the studies in this review, it is also pivotal that future studies include consistent reporting of methodology, including data collection and analysis and intervention outcomes, such as through the use of standard reporting guidelines via the Equator Network [79]. It is necessary to move beyond short-term assessments of program outcomes towards comprehensive, longitudinal evaluations that measure the effectiveness of these health promotion interventions as well as their impacts on long-term behaviour change and health outcomes. Process evaluation of program implementation, including reach, adoption, fidelity of intervention, cost-effectiveness, scalability and community satisfaction, are also warranted for all health promotion programs to ensure sustainability and maintenance of these efforts.

### 4.2. Strengths and Limitations

A strength of this scoping review includes the wide range of information sources, including databases and grey literature. This review was the first to examine health and nutrition promotion programs for communicable diseases and NCDs in PNG. A key limitation is that health and nutrition promotion programs that may have been implemented in PNG but not formally evaluated or recorded in reports or the scientific literature were not captured by this scoping review, which may limit findings, particularly those of newer programs targeted at NCDs. Evidence-based program planning, along with building local research capacity within PNG, could address and mitigate this issue.

## 5. Conclusions

This review has identified an urgent need for health and nutrition promotion programs to target preventative lifestyle health behaviours and NCDs for individuals and communities. This review also provides evidence to support strategies in health and nutrition promotion programs such as cultural appropriateness, mobilising community action and personal skills development through education and training. Effective collaboration through gaining leadership commitment to sustained funding and resources and strengthening international and organisational partnerships will help to activate support for any future programs or policies. Future research should focus on comprehensive, longitudinal evaluations of current or future programs to better the health outcomes of the people of PNG.

## Figures and Tables

**Table 1:** Inclusion and exclusion criteria for the studies.

	Inclusion Criteria	Exclusion Criteria
Study design	All primary study designs, government reports and websites. Studies in humans and in the English language. No restriction on publication date.	Conference abstracts, thesis, systematic reviews, meta-analyses, policy papers, expert opinions and non-peer reviewed articles.
Participants	All participants	Not applicable
Concept	Primary health promotion interventions for non-communicable diseases and communicable disease.	Health promotion policies, such as healthy catering policies, no smoking policies, envi-ronmental policies, or urban design modification as they are not primary interventions.
Context (Setting)	Settings where the majority of the population reside primary health care, schools, workplaces, churches, community centres.	Alcohol or drug rehabilitation live-in facilities and facilities for diagnosed mental illness.
Timing	All interventions lengths.	Not applicable

**Table 2:** Characteristics of included studies and health and nutrition promotion programs in PNG.

First Author, Year	Program Name	Type of Program, Aim/Purpose	Targeted Area	Target Population/Study Population/ Sample Size/Setting/Year	Geographical Region	Study Design	Intervention	Ottawa Charter Key Action Area Addressed
Adepoyibi, 2019 [39]	Daru patient education and counselling model	Training, education, counselling program. Pilot model. To train and educate people living with TB as peer counsellors to reduce TB.	TB	Community. Lead counsellors (*n* = 3) and peer counsellors (*n* = 10) were trained; these provided PEC services to drug-resistant TB patients (*n* = 331). Daru, 2016	Southern	Quantitative.	A pilot model of education and counselling for DR-TB was implemented in Daru, wherein individuals in the community living with TB were trained to be counsellors and peer counsellors. These counsellors delivered sessions and provided novel education resources to drug-resistant TB patients. They also referred patients to appropriate clinicians and other services.	Develop personal skills Strengthen community action
Amini, 2012 [22]	National TB program (from WHO Stop Tuberculosis Program: Directly Observed Treatment, Short course Strategy)	Treatment program—medication. To create a world free of TB, with zero deaths, disease and suffering due to the disease.	TB	Nation-wide population. PNG	All	Program evaluation. Editorial. Qualitative.	Used responsible community members on a voluntary basis to oversee the treatment of TB patients. Patients were provided with fixed-dose combination therapy drugs aimed to reduce pill burden and risk of multidrug resistance. Areas were supplied with high quality TB medications from prequalified suppliers.	Reorientation of health services Strengthen community action
Maha, 2019 [40]	Training, Awareness and Clinical Outreach	Training, patient, public awareness, health service program. To increase community-based care and treatment outcomes of TB.	TB	Health workers, patients attending health services. Patients with presumptive TB were screened (*n* = 3776). TB programs at the Nonga General Hospital, Rabaul Urban Clinic and Kerevat District Hospital in East New Britain Province, 2014–2017	Islands	Retrospective cohort study. Quantitative (data collected from the TB laboratory registers and TB treatment registers at all three facilities).	The decentralisation of TB services from the provincial to the district level by training health workers, raising community awareness about TB and establishing a weekly Clinical Outreach service.	Develop personal skills Reorient health services
Morris, 2019 [41]	Not reported	Diagnosis and treatment program. To describe programmatic interventions for TB in the South Fly District and evaluate characteristics of TB case notifications, drug resistance and treatment outcomes.	TB	Patients attending health services. All patients (*n* = 1548) enrolled on TB treatment at Daru General Hospital, 2014–2017	Southern	Retrospective cohort study. Mixed-method qualitative and quantitative (data was collected from electronic medical records and treatment registers).	To reduce TB transmission, the interventions outlined in the implementation plan were (1) to develop a patient-centred model for diagnosing, treating and preventing drug-susceptible TB and multidrug-resistant, (2) to enhance health systems building blocks by using the WHO framework to facilitate a functional TB care model, (3) to improve utlisation of services through community involvement, (4) to improve service program data for informed decision-making and (5) to establish two decentralised TB care sites in the South Fly District.	Reorient health services
Van Maaren, 2007 [23]	Stop TB Special Project	National project. To halve 2000 levels of TB prevalence and mortality by 2010 through first reaching the global 2005 TB targets. To detect 70% of infectious TB cases and cure 85% by 2005.	TB	Nation-wide population. PNG, 2005	All	Quantitative (case detection rates and treatment success rates were collected for each country targeted).	This project involved expansion of WHO’s Directly Observed Treatment, Short course strategy across the region to cover additional public hospitals and private sectors. A Technical Advisory Group provided strategic guidance including an increased budget for human and financial resources budget to combat TB and increased external funding through partnerships with other governments.	Reorient health services
Burkot, 2017 [24]	Marasin Stoa Kipa program	Training, diagnosis and treatment program. A community-based malaria diagnosis and treatment program (implemented since 2007) aimed to reduce malaria morbidity and mortality by strengthening local health facilities.	Malaria	Community (local women). Program coverage (*n* = 10,850). Participants (*n* = 61). Major oil and gas development area in the Southern Highlands—communities in Lake Kutubu region, 2015.	Highlands	Program evaluation Qualitative (data collected from semi-structured in-depth interviews and focus group discussions through transcription and translation).	Respected women in the community were recruited as medicine storekeepers in their respective villages. They were trained by field officers who were responsible for quality assurance of malaria diagnosis and treatment service delivery. They were issued with kits comprising of the necessary supplies for malaria diagnosis and treatment. Some financial and material incentives were offered.	Develop personal skills Strengthen community action
Fitzpatrick, 2007 [25]	Batri Village Bed Net Initiative (development of Erima Empowerment Research Health Promotion Project)	Education, resource provision program. An empowerment approach to community development to malaria reduce prevalence among remote members of the Kewapi language group in PNG.	Malaria	Community. WHO mosquito nets (*n* = 400). Kewapi language group living in Batri village. Research team included the Kewapi language group living in urban Erima, Port Moresby, 2004	Highlands	Program evaluation. Qualitative.	The initiative focused on community development through empowering members of the Kewapi language group in urban Erima to take responsibility for the obtaining, distributing and ensuring effective use of bed nets in the Batri village. This approach involved discussions with various stakeholders including Batri village Elders and the PNG British High Commission.	Develop personal skills Strengthen community action
Hetzel, 2012 [26]	Nationwide availability of free long-lasting insecticide treated nets	Resource provision program. To achieve 80% household ownership and 80% usage in children under the age of five years and pregnant women with a distribution rate of one long-lasting insecticide treated nets per 2.5 people.	Malaria	Nation-wide population. Over the five-year grant period, long-lasting insecticide treated nets (*n* = 2,321,100) were supplied by Rotarians Against Malaria. PNG, 2001–2009	All	Cross-sectional household survey. Mixed-method qualitative and quantitative (data collected from a structured questionnaire).	Country-wide campaign for distribution of long-lasting insecticide treated nets. The grant endorsed a campaign-based “catch up” strategy which aimed at rapidly achieving high levels of net ownership and utilisation. The long-lasting insecticide treated nets were imported and delivered to provincial or district headquarters where local health authorities were responsible for distribution to the households.	Create supportive environments
Kurumop, 2013 [27]	National malaria treatment protocol	Technology—text messaging program. A text message reminder service designed to support health worker adherence to a revised malaria treatment protocol	Malaria	Health workers. Purposively selected health facilities (*n* = 6) and health workers (*n* = 42) located in the Eastern Highlands Province of PNG, 2011	Highlands	Program evaluation. Mixed-method qualitative and quantitative (data collected from transmission reports, study diaries and complementary health facility survey).	Ten text messages were sent twice (two texts a day from Monday to Friday) to 42 health workers across two-weeks. These served as reminders to paticipants about key points of the protocol. The messages were composed in English.	Reorient health services
Rosewell, 2017 [28]	Online National Health Information System	Technology—Surveillance program. An mobile health initiative to strengthen malaria surveillance in a 184-health facility, multi-province, project aimed at strengthening the National Health Information System in a country with fragmented malaria surveillance, striving towards enhanced control, pre-elimination.	Malaria	Health facilities. Health facility (*n* = 184), multi-province in PNG, 2015	All	Program evaluation. Qualitative (hard-copy and electronic data are collected).	A mobile application with remote loading capacity and an online platform was developed for health professionals. A malaria testing register was established where geocoded households, villages and health facilities were integrated. A malaria program management dashboard featuring village-level mapping tools and statistical algorithms to detect malaria outbreaks was created.	Reorient health services Create supportive environments
Carmone, 2014 [29]	Prevention of parent-to-child transmission of HIV program	Counselling, care, diagnosis and treatment, health service program. To promote uptake of HIV testing and care among partners of HIV-infected pregnant women, to increase program retention and infant HIV-free survival.	HIV	Families attending health services. Women (*n* = 265) who were registered for the program services at Goroka Family Clinic at the Eastern Highlands Provincial Hospital, PNG, 2007–2011	Highlands	Program evaluation. Quantitative (data collection through transcription of paper charts to standardised study forms, using electronic data and clinician interviews to fill any gaps).	During the program, the PNG National Guidelines for HIV Care and Treatment served as the framework for directing clinical care and antiretroviral therapy for pregnant women. As per the Patient and Provider Unified Approach model, the program services adopted a patient-centred and family-focused approach. Included was a case management system where there was ongoing, in-depth patient counselling to address barriers to care, ensuring high quality care. Case managers aimed to establish a trusting, safe and supportive environment for women, encouraging them to disclose their HIV diagnosis to their families and bring their male partners and children for testing and counselling. There were same-day, same-place appointments for antenatal and HIV care for women, HIV-infected male partners, and HIV-exposed infants. Families and women were reimbursed for transportation costs and received modest nutrition support.	Develop personal skills Reorient health services
Kelly-Hanku, 2020 [30]	Mother to child transmission program	Care, diagnosis and treatment, health service program. Prevention of mother to child transmission of HIV through supporting maternal adherence to antiretroviral therapy.	HIV	Families attending health services. Women (*n* = 763) enrolled in the program from in two antenatal clinics in Port Moresby & Goroka (Eastern Highlands Province), 2012–2015	Southern and Highlands	Retrospective clinical audit to investigate factors associated with program performance outcomes including loss to follow-up of pregnant women. Quantitative (data sources included paper-based antenatal records of HIV-positive women and their infants’ medical records).	In Port Moresby, for the first 6 weeks, prevention of mother to child transmission services were integrated in antenatal, delivery and postnatal care services. After 6 weeks, HIV-exposed infants were referred to the paediatric HIV clinic (for HIV prophylaxis, confirmatory testing and treatment) and mothers to the outpatient adult antiretroviral therapy clinic. The HIV clinics operated on different days, were not co-located and records were not linked. In Goroka, services were integrated in antenatal, delivery and postnatal care services, with both mother and infant receiving care from the same clinical team until infant HIV confirmatory testing at 18 months. Infants enrolled in the paediatric clinic at 6 weeks and mother transferred to adult antiretroviral therapy clinic at 18 months. All healthcare was provided by government healthcare workers or staff funded by Australian government.	Develop personal skills Reorient health services
Tynan, 2018 [31]	Prevention of parent-to-child transmission programs	Care, diagnosis and treatment, health service program. Preventing and elimination of neonatal HIV transmission.	HIV	Families attending health services. Health care workers (*n* = 16) from Port Moresby, National Capital District; Mt Hagen, Western Highlands Province	Southern and Highlands	Program evaluation. Qualitative (16 semi-structured interviews with health care workers to examine their perception of health system factors impacting outcomes of transmission programs.) Purposive sampling was used.	Transmission programs are a complex package of health care interventions delivered over time, across stages of women’s pregnancy and the developmental lifespan of children. These services encompass antenatal care, provision of adult antiretroviral therapy, HIV counselling, testing and treatment, infant prophylaxis, and linkages to long-term HIV care.	Reorient health services
Katz, 2007 [32]	Tingim Laip (Think of Life) Mobilisation	Social mobilisation program. HIV prevention through social mobilisation in settings characterised by rapid change and high levels of mobility.	HIV	Community. Estimated reach (*n* = 170,000) in 34 sites that have been identified as ‘high risk areas’ or ‘hotspots’, 2005	All	Program evaluation. Qualitative.	The Tingim Laip Mobilisation is a bottom-up communication for social change initiative aimed at addressing HIV/AIDS in PNG through social mobilisation. Community ‘settings’ were chosen based on epidemiological data (hotspots), willingness to actively participate in the project and the site committee’s capacity to implement activities for HIV prevention. It places emphasis on innovative interventions and ‘action-oriented’ communication. Local communities develop messages according to local customs and needs by using a bottom-up approach.	Strengthen community action
Kelly-Hanku, 2014 [42]	Catholic HIV and AIDS Service Inc. information, education and communicat-ion materials	Print and digital media—Education, public awareness program. The materials aimed to raise awareness and improve attitudes towards HIV and AIDs.	HIV	Community. Questionnaires (*n* = 23): Eastern Highlands, Western Highlands, Madang Key informant interviews (*n* = 13): National Capital District, Eastern Highlands, Southern Highlands, Western Highlands, Enga, West Sepik, East Sepik, Milne Bay Focus groups (*n* = 32): Simbu (Mingende), Jiwaka (Banz), 2014	Highlands, Momase and Southern	Mixed-method qualitative study (Questionnaires key informant interviews, focus groups including one group that was specifically for people living with HIV).	The materials can encompass posters, brochures, flyers, billboards, online/social media, and TV and radio programs. The use of various media formats is important for communicating initiatives and inducing change in attitudes through reaching diverse populations and reinforcing messages through different formats. The messages reflected the organisational values of acceptance, compassion and love. They are designed to be person-centred, encouraging and supportive as opposed to inciting fear. Most of the materials were produced in English.	Develop personal skills
Lupiwa, 1996 [33]	Health education program	Training, education program. To raise awareness about the transmission, treatment and prevention of other sexually transmitted diseases.	HIV (and other sexually transmitted diseases)	Institute of Medical Research staff, community. Rural and peri-urban communities of Asaro Valley near Goroka (Eastern Highlands Province).	Highlands	Program evaluation. Qualitative (informal discussions with people in the communities, individual interviews, experiences when conducting workshops).	Training workshops were conducted for PNG Institute of Medical Research staff. This included visual aid development for health education programs, teaching techniques for lectures, group discussions, individual health education, demonstrations and interviewing skills. Practice sessions were then conducted in various community settings to assess the effectiveness of the different teaching methods and visual aids. Groups were separated by gender and facilitated by a same gender health educator. Visual aids were made with inexpensive, locally available materials. The were used to tell stories and explain technical points. To ensure a comfortable environment, each session was prefaced by explaining the potentially explicit and uncomfortable images, and participants were given the opportunity to leave as they wished. Educational videos were produced in Tok Pisin.	Develop personal skills
Thomas, 2012 [43]	Komuniti Tok Piksa Project	Digital media—education, public awareness program. The project aimed to understand local narratives around HIV/AIDS and translate these into visual education and prevention material in collaboration with communities.	HIV	Community. Baseline study conducted in nine communities (*n* = 118). Film production and screening in the five Highland provinces. 2009–2011	Highlands	Program evaluation. Mixed-method qualitative study (focus groups, individual surveys).	An initial baseline study was conducted through use of in-depth interviews to assess existing knowledge about HIV and AIDS in Highland communities, and to provide a basis for ongoing monitoring of community views and attitudes. These narratives were then taken into consideration for film production. Community members were engaged in a collaborative film-production process to ensure appropriate representation for the stories and maximise potential impact for HIV education.	Develop personal skills Strengthen community action
Thomas, 2016 [44]	Komuniti Tok Piksa Project	Digital media—education, public awareness program. The project aimed to understand local narratives around HIV/AIDS and translate these into visual education and prevention material in collaboration with communities.	HIV	Community. Individual surveys (*n* = 1099), focus groups from screenings held in 110 village cinemas in PNG highlands.	Highlands	Qualitative (individual surveys).	The distribution of the films “as above” was conducted by training community facilitators to host screenings in existing screen venues in the same communities in which they were produced (e.g., in cassette disk houses or ‘haus piksa’).	Develop personal skills Strengthen community action
Camara, 2022 [34]	Self-collect HPV same-day screen-and-treat program	Training, screening, diagnosis and treatment, education program. To increase cervical screening coverage and timely treatment, reducing morbidity and mortality by cervical cancer.	HPV	Health workers, women attending health services. Women who attended the two Well Women Clinics (Madang Province and Western Highlands Province) and participated in the HPV program.	Momase and Highlands	Evaluation of a field trial to examine women’s acceptability of self-collection process and HPV program intervention. Qualitative (62 semi-structured interviews).	Health care workers were employed in a pre-intervention training program where they were trained to carry out same-day HPV program. This included comprehensive health education, applying thermal ablation, and post-test counselling. Health education group sessions (10–15 min) were conducted in clinic with attending women prior to self-collection. Health care workers delivered information on: cervical cancer signs and symptoms, its association to HPV, and instructions on self-collection procedures. The services were free.	Develop personal skills
Tynan, 2012 [35]	No-scalpel vasectomy program	Surgical, health service program. Family planning and addressing population growth in PNG.	Family planning	Nation-wide population. Frontline health care workers (*n* = 7) and upper-level health system officials (*n* = 7) Involved in programs, 2011.	All	Mixed-method qualitative study (14 in-depth interviews with frontline health workers and key government officials, documentary and policy analysis).	The No-scalpel vasectomy program is a component of the National Department of Health family planning program. The decentralised health system means that provincial governments are responsible for administration and prioritisation of the service at a local level.	Reorient health services
Ropa, 2019 [36]	Field epidemiology training programme of PNG	Training program. A National Government initiative to strengthen the public health workforce and health systems across all levels of government.	Health service.	Health workers. The program graduated field epidemiologists (*n* = 81) who completed projects that contributed to improvements in public health practice in PNG, 2013–2018	All	Program evaluation. Qualitative.	The training program encompassed the design, implementation, and evaluation of evidence-based interventions by health workers (Fellows). Five training phases were conducted across 8 months, where Fellows were supported by trainers and mentors consisting of epidemiologists, physicians, and public health managers. Fellows engaged in training workshops and allocate around 10% of their time to working on field-based epidemiology and intervention projects within their usual workplace. The program mainly focused on communicable diseases however, Fellows could choose to conduct field projects related to other public health priorities.	Reorient health services
Chen, 2018 [37]	School educating program for betel quid chewing	Education program. To educate primary and secondary school students in PNG about oral cancer and betel nut chewing.	Betel nut chewing, Oral cancer	Primary and secondary school students. Primary school students (*n* = 95) and secondary school students (*n* = 55). Markham Road primary school, Koiari Park Adventist Secondary School in Lae city, 2015.	Momase	Cross-sectional school-based survey and self-administered questionnaire. Mixed-method qualitative and quantitative.	30-min lectures covering various topics relating to oral cancer, including epidemiology, etiology, clinical presentation, management and treatment. The questionnaire material aligned wth the content presented lectures. Since English was the primary language used at school, it was used for the lectures. Baseline measurements of all students were taken, followed by the lectures and then a post-test to examine their knowledge on oral cancer, betel nuts and tobacco cessation. They were given 15 min to complete the same questionnaire before and after the lectures, which comprised of two sections (knowledge and attitude). Subsequently, a post-education survey was conducted to assess improvements.	Develop personal skills
Rowse, 2013 [38]	The Sugarman education project	Education program. Health-promoting education about T2DM to the Indigenous communities of Bougainville in PNG.	T2DM	Community. Participants (*n* = 2900) in Indigenous communities (*n* = 16) of Bougainville, PNG participated in Sugarman presentations (*n* = 26) over a five-week period, 2010	Islands	Program evaluation. Mixed-method qualitative and quantitative. To evaluate and determine changes in participants’ diabetes knowledge, anonymous pre and post presentation self-adminstered surveys were completed and analysed.	The intervention involved a one-off 40 min ‘Sugarman’ presentation designed to raise awareness of diet as a modifiable risk factor for type 2 diabetes, in particular about hidden sugars in foods and their impact on health. The presentation incorporated audience interaction, visual displays, and a large 6-metre Sugarman representation to illustrate the process of sugar metabolism in a healthy person versus someone consuming excessive high sugar processed foods. The consequences of uncontrolled sugar intake, leading to diabetes and associated complications like blindness, kidney failure, heart attacks, and foot sores, are discussed. The intervention emphasised prevention through healthy lifestyle choices such as exercise, a natural diet consisting of local foods, and not smoking. Additionally, the presentation covered the treatment of diabetes, highlighting the role of proper nutrition, exercise, and medication in managing the condition and for gaining a positive lifestyle and improved health. An interpreter was trained and validated to provide consistent translations of English into local dialect across the 26 presentations.	Develop personal skills

HIV = Human Immunodeficiency Virus; HPV = Human papillomavirus; T2DM = Type 2 Diabetes Mellitus; TB = tuberculosis.

**Table 3:** Key findings of the included studies and health and nutrition promotion programs in PNG.

First Author, Year	Program Name	Program Outcomes	Enablers	Barriers
Adepoyibi, 2019 [39]	Daru patient education and counselling model	The pilot program contributed to increased retention in care. There was a reduced loss to follow-up, from 18% in 2014 to 4% in 2015.	The program empowered people in the community living with TB to become peer counsellors and deliver information to other patients. This reduced burden for clinical staff, as the task of providing drug-resistant TB education and counselling was time-consuming.	The training and mentoring needed for peer counsellors was resource-intensive. Limited resources and skill including adequate infrastructure, electricity and basic computer skills.
Amini, 2012 [22]	National TB program from WHO Stop Tuberculosis Program: Directly Observed Treatment, Short course Strategy	Successful in Daru but not the rest of PNG.	Effectiveness in Daru attributed to significant resource allocation for fixed-dose combination to address the multi-drug resistant TB problem and reducing pill burden. The involvement of volunteer community members as treatment partners.	Lack of accessibility to TB drug supplies locally due to inadequate supplies (fixed-dose combination was only available at hospitals, not health centres). TB patients discharged too early leading to medication non-adherence and relapse. Lack of formal treatment supervision in the community. National commitment needed for a whole of country TB program
Maha, 2019 [40]	Training, Awareness and Clinical Outreach	Increased clinical attendance at TB diagnostic and treatment services. Increase in screened cases (from 1581 to 2195), total registered TB cases (from 678 to 824) and registered cases at decentralised settings (from 209 to 615). Did not significantly improve treatment outcomes (46% pre, 40% post).	Integrated clinical support to decentralised Basic Management Units is considered to be cost-effective. Improved treatment outcomes were associated with health care provider education, patient education, incorporation of incentives, psychological counselling and support, reminders through mobile technology, and patient tracing for missed appointments.	Proximity of services alone did not suffice to improve outcomes. The distance to the service was associated with unfavourable outcomes, indicating the potential need for further decentralisation to the community level (e.g., ward or village).
Morris, 2019 [41]	Not Reported	From 2014 to 2015, treatment success rate for drug-resistant increased from 55% to 86%, and from 70% to 81% for multi-drug resistant TB.	The combination of high-level political commitment, increased provincial, national and aid funding and resource mobilisation for implementation of the response plan, and cooperation among technical partners, community partners and government stakeholders guided by the strong leadership of provincial and hospital authorities.	Delays in mobilising resources and securing funding.
Van Maaren, 2007 [23]	Stop TB Special Project	21% case detection rate and 65% treatment success rate in PNG (2005).	Strong leadership and technical guidance, funding, government/political commitment, effective partnerships and persistent efforts of front-line TB-care workers. Workforces were motivated by increased staff, training opportunities and incentive schemes.	Low political commitment, inadequate staff including insufficient number of adequately trained staff.
Burkot, 2017 [24]	Marasin Stoa Kipa program	Lowered malaria cases.	Medicine storekeepers felt good about helping their community by decreasing malaria cases. Some gift incentives were well received as they were practical and valuable supplies (solar panels, machetes, clothing). The refresher training offered was mostly perceived as a valuable opportunity to enhance their skills and interact with other medicine storekeepers.	Financial incentives were no longer considered enough or worth the time and effort required. The financial model used meant the reduced malaria cases lead to less patients and therefore less profit for medicine storekeepers. Products and running the shop was expensive and time consuming. No allowances provided in training sessions which compounded the opportunity costs. Lack of active community support (financial or in-kind) of local medicine storekeepers, placing burden on family members to help.
Fitzpatrick, 2007 [25]	Batri Village Bed Net Initiative (development of Erima Empowerment Research Health Promotion Project)	There was a significant decrease in the incidence of malaria-related mortality and morbidity in Batri village. There were no deaths from cerebral malaria since the deployment of nets in 2004, marking an improvement from 5 deaths per year previously.	Community involvement of Batri Village Elders and other members. Respect and acknowledgement of cultural context including providing translations and understanding issues, concerns and realities of village life i.e., the project felt culturally appropriate. Sessions were designed to be inclusive to allow for broad participation of community members through deliberate timing.	Not Reported
Hetzel, 2012 [26]	Nationwide availability of free long-lasting insecticide treated nets	Following the campaign, 65% of households owned a long-lasting insecticide treated nets and 80% some form of mosquito net. Overall usage among household members was 33% for long-lasting insecticide treated nets and 44% for any net. Free long-lasting insecticide treated nets distribution increased net ownership but in general, usage rates were low.	In locations where there were sufficient nets available to household members, the distribution led to a substantial increase in net usage.	Program targets were not met due to operational constraints and shortcomings in the distribution process. The primary reasons for the overall low net usage rates were inadequate number of nets and heterogenous distribution. The lack of a major behavior change campaign to accompany the distribution. Malaria awareness efforts did not notably improve throughout the grant period.
Kurumop, 2013 [27]	National malaria treatment protocol	The frequency of the text messages (two per day) was widely acceptable.	The majority of health workers have access to a mobile phone. The morning text message enabled participants to promptly apply the received information into action, as this was a time when they were at work and alert. The repetition of texts mainly considered helpful in reinforcing the message. The simple nature of the text message service was low burden and reduced cost barriers way for broad scale dissemination of information to health workers.	Delays in the transmission of text messages due to poor network coverage often meant participants were receiving them after work hours. Challenges such as lack of phone access or weak mobile network connections especially in rural areas, leaving phones at home, switching off phones to conserve battery, or language difficulties. Beyond the initial two-week pilot period, participants believed that ongoing repetition of the same messages was not desired.
Rosewell, 2017 [28]	Online National Health Information System	Successful in detecting outbreaks, facilitated the monitoring of transmission and mortality, provided timely information on supply requirements, and supported intervention evaluations during outbreaks. Enabled the malaria program management dashboard to facilitate timely monitoring of treatment protocol adherence.	The use of mobile technologies and geographic information system allowed for the timely capturing and reporting of high-quality, geo-coded, case-based malaria data essential for evaluating the scale-up of and sustainability of malaria elimination efforts in PNG.	Delays in reporting, data incompleteness and accuracy need to be considered when designing and evaluating alert algorithms. This is a challenge as it restricts the selection of algorithms to those adaptable to available data rather than assuming high reporting completeness.
Carmone, 2014 [29]	Prevention of parent-to-child transmission of HIV program	Pregnant women or mother-infant pairs with a partner tested for HIV was associated with program retention and HIV-free infant survival. Enabling diagnosis disclosure, partner testing and linkage to care through these programs may help to eliminate paediatric HIV.	Program maturation to be more family-centered (case management intervention to help women disclose their diagnosis to partners and encouraging partner testing), changes in treatment guidelines, and increased provider confidence and experience are potential contributing factors to improved HIV-free survival with time. Better uptake was observed when antiretroviral therapy was provided in the same location as program services, such as in decentralised sites. A greater number of antenatal visits and a longer time on antiretroviral therapy before delivery (>24 weeks) was associated with infant HIV-free survival. Reimbursement received for transportation costs	For successful completion of the programs, women without a partner or a partner who was not tested are key groups that may need additional support. Sufficient drug supply and antiretroviral therapy stockouts presented a barrier to optimal exposure of antiretroviral therapy for pregnant women.
Kelly-Hanku, 2020 [30]	Mother to child transmission program	Across the two well-resourced sites, there was a loss to follow-up of 10% of women before they gave birth and a further 15% of women after birth but prior to infant registration in the paediatric HIV program. Women in the program need to be better supported to improve outcomes. Within 72 h of birth, most newborns (97%) received ARV prophylaxis in Port Moresby compared to two-thirds in Goroka. Paediatric loss to follow-up rates were higher in Port Moresby in comparison to Goroka. Differentiated HIV care is necessary for women of different sociocultural status.	Knowledge of HIV status before pregnancy was associated with increased antiretroviral therapy adherence.	Women who were newly diagnosed, younger (16–20 years old compared to 21–30), primiparous or in a HIV discordant relation were more likely to be loss to follow-up before delivery. Co-location did not mean better outcomes as loss to follow-up rates for mothers before delivery were higher in Goroka than in Port Moresby (perhaps due to long distances to the clinic, lower educational attainment or long wait times). Male engagement was limited by cultural factors, time and financial barriers and cost of transportation.
Tynan, 2018 [31]	Prevention of parent-to-child transmission programs	Efforts towards increasing accessibility and utilisation of the health services remain suboptimal.	Improvements and introduction of biomedical technology for rapid HIV testing and confirmation at the point-of-test significantly improved testing efficiency.	Loss to follow-up due to transport accessibility and costs involved with appointment attendance. Staff experienced challenges following up HIV-positive mothers in the antenatal phase, mainly due to limited staffing or service scope to support this. Facilities’ physical layout impacted effectiveness due to lack of space to offer adequate service or privacy. Health care workers reported inadequate access to specialist staff, inadequate supervision and mentoring of staff, staff shortages, and inadequate access to professional development for training particularly for those outside urban areas. The PPTCT impact at population level is not well understood due to poor communication, inability to collect and share data across the different services. The lack of central leadership and integration of services resulted in poor delivery.
Katz, 2007 [32]	Tingim Laip (Think of Life) Mobilisation	Following one year of implementation, the project shows promising signs of changing community attitudes and behaviours regarding HIV in vulnerable settings in PNG.	The sites that in accordance with the project criteria for ‘settings’ saw change. The bottom-up approach to communication priorities, strategies and messages helped to ensure local meaning was given to messages to support attitudinal, behavioural and social change. Social Mappers (who conducted local interviews, focus groups) contributed to a comprehensive understanding of local contexts which helped with planning and strategic communication mobilisation efforts. Community empowerment increased motivation by letting the community determine how they want to measure change (participatory monitoring and evaluation) and what is most beneficial for their own interventions, capacities and needs. Networking and sharing also inspired new strategies for adoption.	Sites that were selected not in accordance with the project criteria saw challenges in community mobilisation due to lack of project commitment in the community, and site committee members were more interested in personal benefits.
Kelly-Hanku, 2014 [42]	Catholic HIV and AIDS Service Inc. information, education and communication materials	The direct impact of the materials on change within individuals and communities could not be assessed due to lack of information on distribution of materials. Existing materials were in high demand and generally positively received because of the encouraging and supportive messages.	Focus group participants viewed the messages positively as they were ‘welcoming’. Posters after 2008 which contained fewer words (<20 words) increased message clarity and messages were better recalled. Larger font size was favourable. Participants positively engaged with symbolic or abstract images when tasked to interpret messages.	Text heavy, printed materials were problematic for most people, particularly in low literacy settings. Resources were not necessarily tailored to a defined target audience. In most regions, there was an overwhelming preference for materials to be produced in Tok Pisin. Lacked a process for coordination and monitoring of distribution which is important for documentation of product reach. Production and printing of the materials was also not sustainable over time due to lack of planning and limited human and financial resources.
Lupiwa, 1996 [33]	Health education program	Prior to the education session, majority of women had minimal knowledge about sexually transmitted diseases. After the health education and interviewing, nearly all women consented to vaginal examination. It was important to have dedicated educators who were responsive to community concerns and fostered a comfortable environment for discussion.	Training workshops and practice sessions were helpful for training staff in different teaching methods. This allowed for greater flexibility in the workshops to ensure participant understanding of key points. Line drawings were the most effective and convenient for visual aids. Small single-gender groups with same-gender health educators facilitated a more relaxed environment to discuss sensitive subjects such as sexual attitudes and beliefs with minimal embarrassment. Discussion of learning materials with village leaders to ensure they were appropriate brought increased community enthusiasm and cooperation. The loosely structured sessions gave opportunities for frequent questions and discussion, giving way for a more informal atmosphere.	Cardboard models and puppets were less convenient relative to line drawings due to longer preparation time and technical problems which created distraction.
Thomas, 2012 [43]	Komuniti Tok Piksa Project	The films had success in: promoting HIV prevention using culturally appropriate and community-tailored methods, mobilisation of communities to actively seek more information about HIV and AIDS, encouraging audiences to share practical strategies to address HIV/AIDS challenges, encouraging participants to discuss their own risk behaviours and show willingness to change them, prompting community members’ desire to share and promote the messages to others.	Extensive data collection methods (interviews, focus groups, and filming of individuals enacting their daily lives and discussing it) for the initial baseline study to capture community knowledge, behaviours and attitudes. Films using tailored language to suit the community’s interests, understandings and constraints. Community involvement in film production encouraged dialogue and reflection regarding HIV/AIDS prevention. Training of community facilitators to deliver the program sustainably in the community	Not Reported
Thomas, 2016 [44]	Komuniti Tok Piksa (KTP) Project	It is important to understand local behaviours and perspectives around media consumption. Due to lower levels of literacy, rural areas require a more localised approach to media that is tailored to their culture and language. 90% said that the films changed their view towards people living with HIV/AIDS and 94% said they want to share the films with others. The most common learning outcome was how to accept, care for and support those with HIV/AIDS. The second most common outcome was understanding to take preventative measures for reducing risk of HIV contraction (being faithful to one partner, getting blood tests).	Communities were readily accepting of the localised messages when recognising their own culture and environment in stories told by other people from PNG and film broadcast via the *haus piksa* was able to extend the reach of the messages. Innovative ways of using media presented an opportunity to evoke empathy through the films, which had the strongest influence on HIV destigmatisation and understanding of how to support and care for those with HIV/AIDS.	*Haus piksa* as a site was challenging for researchers at times due to expectations by youth to see other movies.
Camara, 2022 [34]	Self-collect HPV same-day screen-and-treat program	There was a high level of acceptability for the same-day HPV screen-and-treat program amongst PNG women in a high burden, low-resource setting. The availability of self-collection option could potentially increase screening uptake. Most women trusted the testing technology and the yielded results.	For most women, the comfort of having a private space for self-collection increased their willingness to attend the screenings, particularly since sexual and reproductive health can be considered as taboo in PNG. The brush used was described as comfortable which increased comfort and ease. Majority expressed that the healthcare workers were knowledgeable, understanding, and giving of their time and that a positive patient-provider relationship was an influential factor for women sharing their program experiences with others in their communities. All women agreed that the health education sessions were useful in equipping them with the knowledge to perform self-collection and accept treatment. Majority of women thought the self-collection process required minimal effort and the same-day program was perceived as very time efficient. The no-cost service improved access for all women, particularly those who were unemployed or living in remote villages.	Some preferred clinician-collected screening due to lack of confidence in their ability to correctly carry out the self-collection process, and perception that women could potentially manipulate their test results.
Tynan, 2012 [35]	No-scalpel vasectomy (NSV) program	There was mixed outcomes across PNG due to various reasons—competing provincial and district-level priorities, inconsistent support from government departments, staff retention difficulties, inconsistent delivery of training programs.	Service sustainability was heavily dependent on focused leadership and open communication among stakeholders (national government, NGOs, the community). NGOs helped to provide financial or technical support for outreach services or training programs. Community engagement was integral for a successful NSV program.	Frontline health workers mentioned poor support from government including lack of funds and resources, difficulties with transportation and staffing to support NSV activities such as supervision and training of new health workers. High demand and increasing waitlists due to inflexible service times and erratic financial support for staff led to community dissatisfaction.
Ropa, 2019 [36]	Field epidemiology training programme of PNG	Many training program graduates participated in interventions that led to successful public health outcomes, yielding tangible impacts locally. Their skills will further contribute to the advancement of public health in PNG. High retention of graduates within the government system was a key outcome in supporting the goals of strengthening health systems and building a strong network of public health experts.	Central factors for the program’s sustainability and success were the strong support from the National Department of Health in designing, delivering and advocating of the program, as well as the usage of the produced data (policy briefs and stakeholder symposium) and recommendations by Fellows (health care workers) for scaling and sustaining successful interventions. The program was tailored to PNG’s needs and capacity. The training approach empowered Fellows to take charge of local health challenges and take action based their gathered data. Government recognition of the program’s values and the practical recommendations produced from the field projects was a kay factor for retention of Fellows within the government system.	The intervention projects needed to be completed during training and not always continued after graduation. This limited the project’s public health impacts.
Chen, 2018 [37]	School educating program for betel quid chewing	Following the education sessions, overall knowledge of betel quid improved in both the middle school and secondary school groups, and was statistically significant for secondary school students (mean difference 0.700 ± 0.277, 95% CI 0.164–1.248, *p*-value = 0.018)). Despite this, there was no increase in motivation to cease betel nut chewing across both groups.	The program was a cost effective method for improving knowledge of betel quid chewing and its associated health impacts.	The education program was not tailored accordingly to the audience ie. the design could be improved to better engage primary school students. The lectures were too short for most students as it was likely their first introduction to the adverse health effects of betel nut chewing. To improve outcomes, the program should be designed to include parents. The wording of some questions caused confusion and misunderstanding, resulting in unfavourable knowledge outcomes. Use of English was a potential barrier as it was a second language for most students.
Rowse, 2013 [38]	The Sugarman education project	There was an overall demonstrated improvement in participants’ understanding of the prevention, causes, and symptoms T2DM. There was especially a significant increase in knowledge of causes and symptoms in communities that had little pre-existing knowledge about diabetes.	The improved understanding of T2DM may be attributed to the use of a culturally sensitive, interactive framework for this education program. The information provided becomes more meaningful to participants when utilising culturally relevant approaches for discussion of risk behaviours for chronic disease.	Limitations for the project included a low return rate of pre- and post-surveys so affecting the sample of data used in program evaluation; limited facilities in many villages which resulted in the sessions being held in varied locations/settings thus increasing the risk of participant attrition, and unexpected large audiences which posed challenges in survey collection and retrieval. Additionally, the project’s direct impact on improving health outcomes is unknown as this necessitates long-term education and continuous iteration. There is also a lack of infrastructure in Bougainville to accurately measure quantitative changes in blood sugar levels (HbA1c).

HbA1c = Glycated haemoglobin; HIV = Human Immunodeficiency Virus; HPV = Human papillomavirus; T2DM = Type 2 Diabetes Mellitus; TB = tuberculosis.

## Supplementary Materials

The following supporting information can be downloaded at: https://www.mdpi.com/article/10.3390/nu16131999/s1, Table S1: Medline Search Strategy [19].
